# Food Security, Conflict, and COVID-19: Perspective from Afghanistan

**DOI:** 10.4269/ajtmh.21-1058

**Published:** 2021-11-10

**Authors:** Zarmina Islam, Diana Mutasem Kokash, Maryam Salma Babar, Utkarsha Uday, Mohammad Mehedi Hasan, Sudhan Rackimuthu, Mohammad Yasir Essar, Arash Nemat

**Affiliations:** ^1^ Dow University of Health Sciences, Karachi, Pakistan;; ^2^ Dubai Health Authority, Dubai, United Arab Emirates;; ^3^ West Bengal University of Health Sciences, Kolkata, West Bengal, India;; ^4^ Department of Biochemistry and Molecular Biology, Faculty of Life Science, Mawlana Bhashani Science and Technology University, Tangail, Bangladesh;; ^5^ Father Muller Medical College, Mangalore, Karnataka, India;; ^6^ Kabul University of Medical Sciences, Kabul, Afghanistan

## Abstract

Afghanistan, a country challenged by war and conflicts, has been in a state of turmoil for several years. The prolonged suffering has brought many challenges to the country’s inhabitants. Among these, food security is one important cause for concern. Food security occurs when people continuously have physical and economic access to adequate, safe, and nutritious food to meet their dietary requirements and food preferences for a functional and healthy life. Amid the pandemic, Afghanistan has witnessed a large increase in food shortages due to its dependence on neighboring countries. In light of current circumstances, food insecurity, coupled with political instability and the third wave of the COVID-19, have made it extremely hard for people to access daily provisions. Hence, people are left to navigate the COVID-19 pandemic with economic recession and poverty as the backdrop of the other health crises. To mitigate food security, international attempts are the required at this critical juncture. The aim of this article is to understand the causes leading to food insecurity and its implications in Afghanistan and to propose solutions that will improve the overall food security at the policy and implementation levels.

## INTRODUCTION

**Figure 1.**
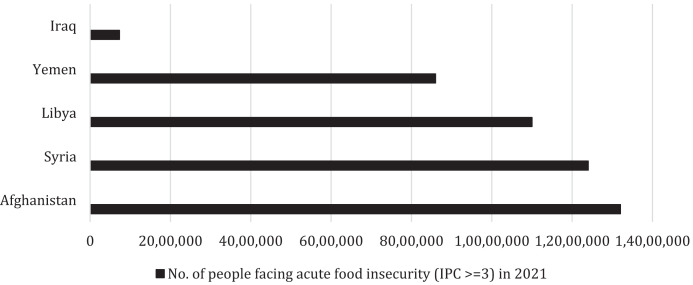
Bar graph depicting number of people facing acute food insecurity (Integrated Food Security Phase Classification [IPC] ≥ 3) in 2021 among other war-torn countries during the COVID-19 pandemic. Sources: Food and Agriculture Organization of the United Nations, April 2021. *Monitoring Food Security in Countries with Conflict Situations*. Available at: https://www.fao.org/3/cb5254en/cb5254en.pdf. World Food Program, May 24, 2021. *WFP Ramps Up Support to Yemen’s Famine Risk Areas*. Available at: https://www.wfp.org/news/wfp-ramps-support-yemens-famine-risk-areas.

Food security occurs when people continuously have physical and economic access to adequate, safe, and nutritious food to meet their dietary requirements and food preferences for a functional and healthy life.^1^ Despite global efforts and attempts to decrease poverty in Afghanistan, the country is still suffering from economic recession. The combined effects of years of conflict, the impact of drought due to climate change, and the COVID-19 pandemic have aggravated food insecurity.^2^ According to the Integrated Phase Classification (IPC), it is projected that 14 million people in Afghanistan are experiencing food insecurity. Poverty in Afghanistan is strikingly high, with more than half of Afghanistan’s 30 million people living below the poverty line and approximately 11 million Afghans facing severe food insecurity^3^ (Figure 1).

Over the past few decades, war and internal conflict have compromised Afghanistan’s public infrastructure.^4^ On August 15, 2021, the Taliban took control of the capital, Kabul, after the withdrawal of US and NATO troops was announced. Since then, the country’s political situation has become a humanitarian crisis.^5^^,^^6^ Monetary aid, which contributed to 70% of government funding, is likely to decrease under the Taliban’s control.^6^

The escalating political circumstances are occurring in the context of the emergence of the Delta strain of the coronavirus in Afghanistan, which has caused a surge in the number of active cases. Sixty percent of reported cases in Kabul were due to the Delta strain.^4^ As of October 17, 2021, there were 155,765 total cases of COVID-19 and 7,243 COVID-19 deaths reported in Afghanistan; however, numbers are likely to be higher due to the shortage of testing centers.^7^ COVID-19 exacerbates the preexisting food crisis with government restrictions and lockdown measures put in place, limiting available job opportunities and income.^8^ This article aims to provide a critical commentary on the cumulative impacts of food insecurity, conflict, and COVID-19 on Afghanistan and offer recommendations on how to improve the situation.

## CAUSES LEADING TO FOOD INSECURITY IN AFGHANISTAN

According to the United Nations, nearly 12 million Afghan citizens face acute food insecurity and lack access to stable jobs and income.^9^ One of the most extreme effects of the pandemic is the economy contraction of 1.9% and the increase in poverty levels from 41.6% to 45.5%, thereby exacerbating food insecurity.^10^ Loss of employment (36%), reduced income (18%), severe sickness or death of breadwinners due to COVID-19 (12%), hikes in food prices (11%), and severe sickness or natural death of breadwinners unrelated to COVID-19 (8%) are impacts that households have experienced during the pandemic.^11^

Global restriction applied to mitigate the spread of a second wave of COVID-19 led to a macroeconomic downturn in Afghanistan, ultimately affecting access to food and other basic needs due to closing of borders. As a result, in March 2020, closure of routes to and from suppliers in Pakistan—one of Afghanistan’s primary food import and export sources—caused a food shortage.^10^ Inaccessibility to food markets and road blockages in areas such as Daikundi province, the city of Bamyan, and Ghor, Badakshan, and Nuristan provinces, as well as a few districts in the north of the country, likely limited people’s access of people to food due to snowfall during the winter season.^11^ Inflation began to rise in early 2021 as strengthening oil prices increased food transportation costs by as much as 50% in some areas. However, its impact will be larger in areas with access challenges, particularly for the hard-to-reach and high-elevation areas that experience cold winters. Poor pasture conditions during flash floods, snowfall in high altitudes, and lack of access to crop residual fodder make livestock conditions worse, resulting in their poor physical condition and low milk productivity and consequently affecting both income and nutrition from the livestock sector.^11^

Afghanistan faces one of the world’s most acute internal displacement crises because it suffers from prolonged conflict, ongoing insecurity amid the pandemic, and natural calamities. Afghanis often migrate without the necessary legal documents, hindering their access to support services.^11^ It is now estimated that, after the Talibanis regained control of the country, 14 million people, including two million children—approximately one in three people in the country—are food insecure. Since August 2021, the nation has faced potential economic collapse, as well as a likely increase in the number of people living below the poverty line. The cost of essential goods has doubled within the brief span of the Taliban’s military campaign and is expected to continue to rise with inflation. According to Reuters, water sold at the Kabul airport is now costing as much as USD 40 a bottle. Monetary aid that used to be provided by other nations—the source of an estimated 75% of the country’s government funding—is likely to disappear under Taliban control. This lack of aid, paired with exponentially increasing food prices, is likely to leave millions hungry.^6^ After the decisions of Western Union and MoneyGram to suspend services in the country, many Afghans confront these rising prices without the crucial support of funds sent by family members abroad. Local warlords also hoarded many shipments, causing riots.^6^ These factors ultimately led to the vicious cycle of food insecurity, and urgent solutions are required.

## IMPLICATIONS OF FOOD INSECURITY IN AFGHANISTAN

Food insecurity is one of the most severe socioeconomic and public health issues affecting low- and middle-income countries. It is linked to poorer health outcomes^12^ and self-reported health status; depression and anxiety;^13^ reduced micronutrient, fruit, and vegetable consumption;^14^ obesity and overweight; poor child growth;^15^ and birth abnormalities.^16^ Those affected by poverty or food insecurity are likely to have additional resource-related difficulties (e.g., housing insecurity, energy insecurity), which can worsen poor nutrition, health, and illness management. People who live in or near poverty have disproportionately poorer outcomes and reduced access to health care than those who do not.^17^ Food insecurity can aggravate existing conditions, for instance, poor glycemic control in patients with diabetes, end-stage renal illness in chronic kidney disease patients, and low CD4 counts and poor antiretroviral medication adherence in HIV patients. Food insecurity can also exacerbate health issues and costs encountered by households with children who have special healthcare needs or individuals with disabilities.^18^

In addition to food shortages, the country is currently experiencing a severe drought, causing crop outputs to plummet. In a country where agriculture accounts for a quarter of gross domestic product, these losses are likely to have a devastating impact on the economy and result in job loss.^6^ Crop failure is a significant source of food insecurity for Afghans living in rural areas. Farm families who are forced to relocate lose access to their traditional sources of food, increasing their struggle to find a decent meal. Drought and irregular rainfall patterns during the most recent planting season are also predicted to reduce the country’s autumn wheat yield by 20% to 30%. Wheat, the country’s main cereal crop, is used to make bread, a staple of rural diets.^6^ In 2021, 36% of the Afgani population was classified as facing crisis (IPC Phase 3) or emergency (IPC Phase 4) compared with 33% in 2020.^11^

## EFFORTS AND RECOMMENDATIONS

Several efforts to mitigate this crisis are underway with a focus on diversification of the economy, provision of basic needs to disadvantaged communities in rural areas, and a balance between health measures and economic implications. Such efforts have been implemented in Africa^20^ and Afghanistan in the past; however, new barriers, including strict border control, suspension of flights, and civil unrest, have complicated access. Hence, initiatives to cooperate with neighboring nations are underway.^6^^,^^8^^,^^9^

The World Food Program (WFP) of the United Nations (UN) has been delivering food relief on the ground in Afghanistan for more than 60 years, with more than 5.5 million people receiving help in the first half of 2021.^19^ Their help has gone toward strengthening local food systems, which includes helping local farmers and businesses as well as boosting community infrastructure, paying special attention to mothers and children, who are particularly vulnerable to malnutrition.^6^ Despite a history of refusing to allow the UN to function in areas held by the Taliban, the WFP has indicated that it will continue to operate unhindered in the country. However, the UN warned that if emergency help does did come by September, Afghanistan could run out of food. More than 550 tons of emergency first-aid supplies and malnutrition kits have already been dispatched to Afghanistan but are still in transit due to the suspension of commercial flights to Kabul airport. Food aid will continue to be delivered by truck, according to the WFP.^6^ In addition, 750,000 Afghan households (5 million people) were provided food necessities by the World Bank in 2021 in conjunction with its Dastarkhwan-e-Meli program, which serves more than 2.2 million people. The program under Community Development Council funded by the COVID-19 Relief Effort for Afghan Communities and Households and the Citizens’ Charter Afghanistan Project is addressing critical food needs in vulnerable communities by providing 2 to 3 weeks of rations. Moreover, a grievance redressal mechanism has been introduced to resolve and discourage instances of corruption in food distribution.^9^ Besides, a grant of USD 97.50 million approved by the World Bank in February 2021 aims to support Afghans challenged by recent droughts and COVID-19 and also via the Early Warning, Early Finance and Early Action Project.^10^

Action plans should aim to provide farmers in rural areas with quality seeds and assurance of humanitarian aid to prevent economic downfall.^11^ Moreover, collaborative efforts to legalize opium production for medical purposes through discussion with neighboring nations and Taliban stakeholders is necessary to maintain livelihood in rural areas.^6^ More important, creating opportunities for vulnerable people such as those with unsustainable incomes, households supported by women, and individuals with disabilities through short-term aid as well as entrepreneurial-focused interest-free micro-loans are recommended. Supplementing this with training workshops on entrepreneurship, budgeting, and a potential youth savings program can equip civilians with the skills to manage their finances during a crisis. Continual monitoring during this harvest season is crucial, and hence crop pest and livestock diseases must be addressed immediately.^11^ In urban areas, smart local strategies to capitalize on skills-based services, promotion of local markets, and encouraging those outside of Afghanistan to invest in and support Afghan businesses is important.^11^ Cooperative efforts with neighboring Pakistan to both establish food warehouses and create accessible aid opportunities is necessary with current border constraints. Lastly, creation of job opportunities in other sectors, especially mineral production such as copper, must continue to promote economic prosperity. These recommendations, coupled with international attempts to ease tensions, increase refugee support services, and purchase of Afghan goods and services is vital to resolving food insecurity.

## References

[1] Food and Agriculture Organization of the United Nations , 2021. Food security. *Policy Brief*, No. 2. Available at: http://www.fao.org/fileadmin/templates/faoitaly/documents/pdf/pdf_Food_Security_Cocept_Note.pdf.

[2] United Nations World Food Programme (WFP) Available at: https://www.wfp.org/. Accessed October 4, 2021.

[3] U.S. Agency for International Development *Food Assistance Fact Sheet—Afghanistan*. Available at: https://www.usaid.gov/afghanistan/food-assistance. Accessed October 4, 2021.

[4] Essar MY Hasan MM Islam Z Riaz MMA Aborode AT Ahmad S, 2021. COVID-19 and multiple crises in Afghanistan: an urgent battle. Confl Heal 15: 70. 10.1186/s13031-021-00406-0 PMC8447801 34535160

[5] Ryan M DeYoung K, 2021. Biden to withdraw U.S. forces from Afghanistan by Sept. 11, 2021. *The Washington Post*. Available at: https://www.washingtonpost.com/national-security/biden-us-troop-withdrawal-afghanistan/2021/04/13/918c3cae-9beb-11eb-8a83-3bc1fa69c2e8_story.html. Accessed October 4, 2021.

[6] NYC Food Policy Center (Hunter College) , 2021. *Food Insecurity in Afghanistan*. Available at: https://www.nycfoodpolicy.org/food-insecurity-in-afghanistan/. Accessed October 4, 2021.

[7] Worldometer, 2021. *Afghanistan COVID: 155,309 Cases and 7,214 Deaths.* Available at: https://www.worldometers.info/coronavirus/country/afghanistan/. Accessed October 4, 2021.

[8] Global Platform IPC, 2020. *COVID-19 Exacerbates Afghanistan’s Food Crisis, 10 Million Acutely Food Insecure*. Available at: http://www.ipcinfo.org/ipcinfo-website/ipc-alerts/issue-21/en/. Accessed October 4, 2021.

[9] World Bank , 2021. *Food Relief for Poor Afghans Amid COVID-19*. Available at: https://www.worldbank.org/en/news/feature/2021/05/04/food-relief-for-poor-afghans-amid-covid-19. Accessed October 4, 2021.

[10] Sharma R, 2021. *The Impact of COVID-19 on Poverty in Afghanistan*. The Borgen Project Available at: https://borgenproject.org/the-impact-of-covid-19-on-poverty-in-afghanistan/. Accessed October 4, 2021.

[11] IPC Acute Food Insecurity Analysis , 2020. *COVID-19 Impacts, High Food Prices, Reduced Income and Conflict are Key Drivers of Acute Food Insecurity*. Available at: https://www.fsinplatform.org/sites/default/files/resources/files/IPC_Afghanistan_AcuteFoodInsec_2020Aug2021March_report.pdf.

[12] Ashiabi GS O’Neal KK, 2007. Children’s health status: examining the associations among income poverty, material hardship, and parental factors. PLoS One 2: e940. 17895981 10.1371/journal.pone.0000940 PMC1978512

[13] Hadley D Tegegn A Tessema F Cowan JA Assefa M Galea S, 2008. Food insecurity, stressful life events and symptoms of anxiety and depression in east Africa: evidence from the Gilgel Gibe growth and development study. J Epidemiol Community Health 62: 980–986. 18854502 10.1136/jech.2007.068460

[14] Rao S 2001. Intake of micronutrient-rich foods in rural Indian mothers is associated with the size of their babies at birth: Pune Maternal Nutrition Study. J Nutr 131: 1217–1224. 11285330 10.1093/jn/131.4.1217

[15] Mutisya M Kandala N Ngware MW Kabiru CW, 2015. Household food (in)security and nutritional status of urban poor children aged 6 to 23 months in Kenya. BMC Public Health 15: 1052. 26463345 10.1186/s12889-015-2403-0 PMC4605131

[16] Carmichael SL Yang W Herring A Abrams B Shaw GM, 2007. Maternal food insecurity is associated with increased risk of certain birth defects. J Nutr 137: 2087–2092. 17709447 10.1093/jn/137.9.2087 PMC2063452

[17] Urban Institute , 2015. *How Are Income and Wealth Linked to Health and Longevity?* Available at: https://www.urban.org/research/publication/how-are-income-and-wealth-linked-health-and-longevity. Accessed October 4, 2021.

[18] O’Malley JA Klett BM Klein MD Inman N Beck AF, 2017. Revealing the prevalence and consequences of food insecurity in children with epilepsy. J Community Health 42: 1213–1219. 28477050 10.1007/s10900-017-0372-1

[19] World Food Programme , 2021. *Afghanistan: WFP Committed to Averting Humanitarian Crisis as One in Three People go Hungry.* Available at: https://www.wfp.org/stories/afghanistan-wfp-committed-averting-humanitarian-crisis-one-three-people-go-hungry. Accessed October 6, 2021.

[20] Mohamed EMA Abdallah SMA Ahmadi A Lucero-Prisno DE, 2021. Food security and COVID-19 in Africa: implications and recommendations. Am J Trop Med Hyg 104: 1613–1615. 33684060 10.4269/ajtmh.20-1590 PMC8103487

